# When the Diaphragm Deceives: Subpulmonic Effusion Flagged by AI and Confirmed by Ultrasound

**DOI:** 10.7759/cureus.103161

**Published:** 2026-02-07

**Authors:** Sergio Miravent, Sofia M Silva, Manuel J Navarro, Manuel D Lobo, Rui Pereira de Almeida

**Affiliations:** 1 Department of Basic Emergency Service, Vila Real de Santo António Health Center, Algarve Local Health Unit, Vila Real de Santo António, PRT; 2 Department of Medical Imaging and Radiotherapy, Higher School of Health Sciences, University of Algarve, Faro, PRT; 3 Department of Medical Imaging and Radiotherapy, School of Health Dr. Lopes Dias, Castelo Branco, PRT; 4 Department of Radiology, Local Health Unit of the Northeast, Mogadouro, PRT; 5 Department of Health, Medicine and Life Sciences, University of Luxembourg, Esch-sur-Alzette, LUX

**Keywords:** artificial intelligence, pleural effusion, subpulmonic pleural effusion, thoracic radiography, ultrasonography

## Abstract

Chest radiography remains an important exam for the initial assessment of suspected thoracic disease, including screening and orientation of patients in urgent care settings without on-site specialty coverage. In parallel, there has been a growing adoption of artificial intelligence to assist in the interpretation of chest radiographs; while not diagnostic on its own, it functions as an important clinical adjunct for prioritization and decision support. We report a case in which an artificial intelligence system correctly flagged a bilateral pleural effusion but assigned a lower probability to the right side because a subpulmonic distribution simulated hemidiaphragm elevation and apparently preserved the costophrenic angle on the posteroanterior projection, thereby lowering algorithmic confidence. Screening thoracic ultrasound confirmed a right subpulmonic effusion. The extent of pleural fluid was assessed visually (ocular estimation) and documented accordingly, explaining the apparent asymmetry despite bilateral pleural effusions.

This multimodal correlation prevented misclassification as isolated diaphragmatic elevation and informed subsequent management. This case illustrates a recognizable, discreet pitfall for artificial intelligence systems trained to detect more typical meniscus patterns and underlines the value of targeted ultrasound when radiographic signs are subtle, atypical, or discordant with clinical suspicion. A practical implication stems from the fact that when chest radiography suggests apparent elevation of the hemidiaphragm with a non-obliterated or partially maintained costophrenic angle, physicians should actively consider a subpulmonary distribution and confirm it with thoracic ultrasound, instead of relying on chest radiography alone, even when supported by AI.

## Introduction

Despite major advances in medical imaging, conventional chest radiography (CXR) remains a crucial exam for the initial assessment of suspected thoracic and pulmonary disease [1,2]. Conventional radiography remains widely available and cost-effective.

Although several non-radiologist healthcare professionals have demonstrated measurable ability to interpret chest radiographs, performance remains variable and improves with targeted training; however, accuracy may still be limited in more complex cases without additional education and ongoing support [3-5]. In parallel, there has been a recent trend toward applying artificial intelligence (AI) to the interpretation of chest radiographs, not to replace clinical judgment, but as an adjunct to support interpretation, triage, and decision-making [6-11]. This tool is particularly valuable in peripheral emergency departments far from central hospitals, where urgent shifts are often staffed by general practitioners without immediate access to specialist medical services, and where radiologists are based exclusively at the central hospital and are not available on-site. In the present case, no radiologist report or over-read was obtained; therefore, AI was used strictly as decision support, and thoracic ultrasound was performed as a confirmatory modality when CXR findings were subtle or equivocal. Even in central hospitals, specialist services may face constraints due to staff shortages and overloaded shifts. Accordingly, AI-based systems have been evaluated as decision-support tools for chest radiographs, particularly for screening workflows and clinical decision support [12-14].

AI chest X-ray readers learn patterns from large training datasets. When real-world cases differ from those patterns because findings are rare or atypical, pathologies co-occur in unusual ways, images contain artifacts, or acquisition differs across equipment and protocols, a "domain shift" occurs, meaning a mismatch between the data distribution on which the model was trained and the images encountered in practice. This can increase errors and lead to false negatives or positives or misleading highlights [15-17]. Thus, AI should serve as an adjunct to clinical interpretation, not a replacement [18].

The AI system described in this case report (Lunit INSIGHT CXR; Seoul, Republic of Korea: Lunit Inc.) works by applying an artificial intelligence tool to a chest radiograph, generating an automated assessment that includes a list of evaluated findings and their suggested location on the image. In this interface, the set of radiographic findings that can be assessed comprises atelectasis, calcification, cardiomegaly, consolidation, fibrosis, mediastinal widening, pulmonary nodule, pleural effusion, pneumoperitoneum, pneumothorax, and a dedicated tuberculosis (TB) screening output. For each finding, the interface reports a threshold (Thr), i.e., the predefined decision cut-off used by the software to determine whether a given finding is displayed as flagged (reported and visualized) or instead shown as low confidence.

In one output view, the software overlays elliptical regions of interest on the radiograph, highlighting areas considered most consistent with each suspected abnormality; each ellipse is accompanied by a score or percentage (0-100) reflecting the model’s estimated likelihood for that finding. In a second view, results are shown as a color-coded heatmap superimposed on the radiograph - warmer colors (e.g., yellow/red) indicate higher contribution or predicted probability for a given finding, whereas cooler colors (e.g., green/blue) indicate lower likelihood. These visual overlays are intended as an explainability aid, indicating where the model focused its analysis rather than providing anatomical segmentation or diagnostic confirmation, and they should be interpreted in conjunction with expert human review and clinical correlation.

## Case presentation

An 87-year-old male patient presented to a basic emergency service (BES) with complaints of dyspnea lasting for three days, with worsening symptoms on the day of admission. According to the Manchester Triage System, he was classified as orange; very urgent, based on the "dyspnea" flowchart and the discriminator "speaks in short sentences." Initial vital signs revealed a peripheral oxygen saturation of 96% while breathing room air.

His past medical history included arterial hypertension and self-reported "respiratory problems," which he was unable to specify, and he was unaware of his regular medication. On clinical examination, the patient was in good general condition, alert and oriented, afebrile, eupneic, well-perfused, and well-hydrated. Pulmonary auscultation revealed a global decrease in breath sounds with expiratory wheezes. Cardiac auscultation showed a regular rhythm without murmurs, and abdominal examination was unremarkable, with a soft, depressible, and painless abdomen without signs of peritoneal irritation.

**Figure 1:**
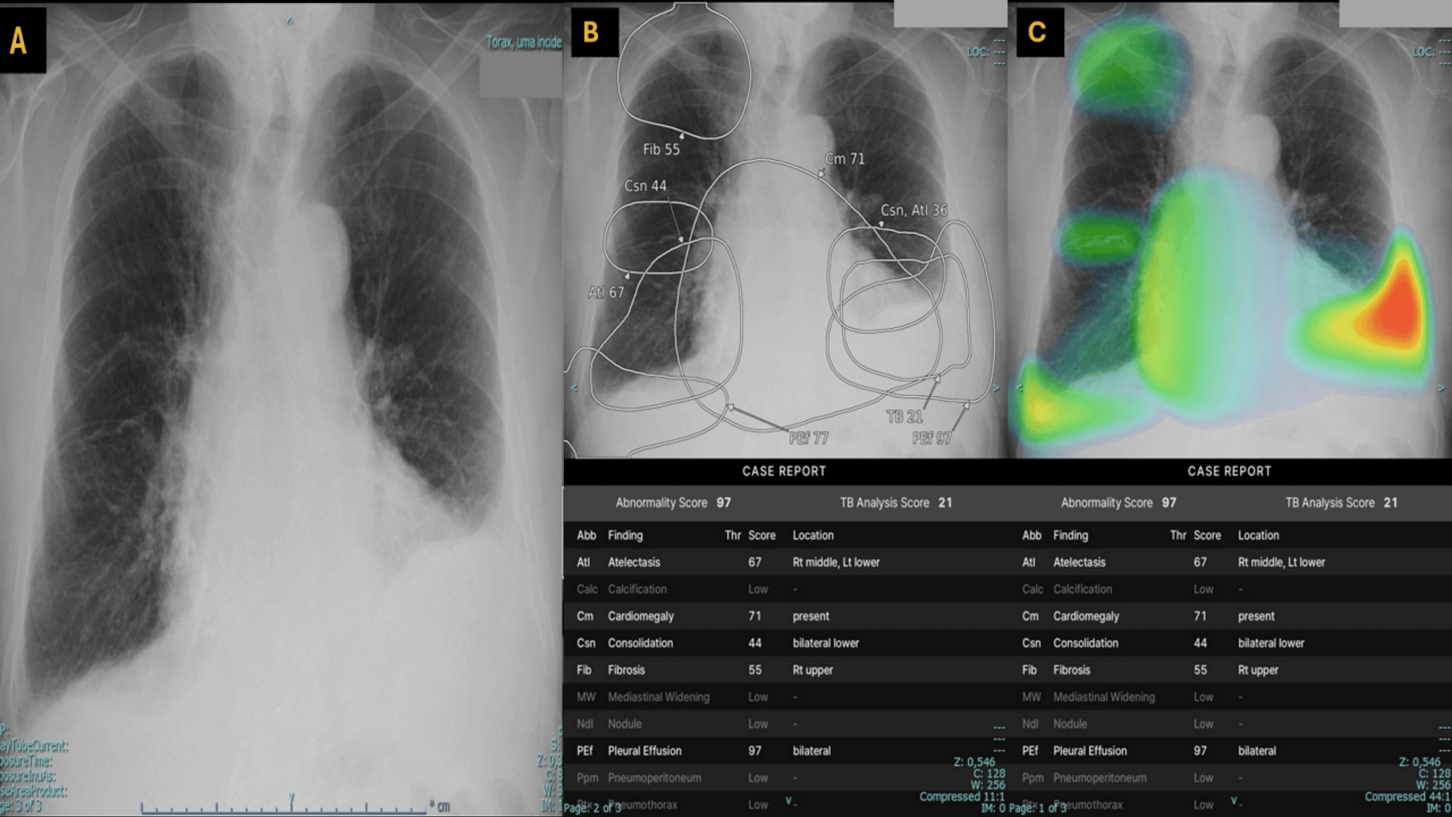
Chest radiograph and AI visual outputs demonstrating possible bilateral pleural effusion with right-sided subpulmonic underestimation. (A) Posteroanterior chest radiograph showing bilateral pleural effusions, with left-sided predominance. The left effusion demonstrates a classic meniscus sign. On the right, there is subtle apparent hemidiaphragm elevation with a poorly defined costophrenic angle and no typical meniscus, suggestive of a possible subpulmonic effusion. (B) AI ellipse overlay on the chest radiograph indicates bilateral pleural effusion, with higher confidence on the left (97%) than on the right (77%). Additional AI-flagged findings include cardiomegaly (71%), atelectasis (67%), lower-zone consolidation (44%), and right upper-lobe fibrosis (55%). (C) AI heatmap overlay shows predominant basal activation, with high-intensity "warm" colors (red/orange/yellow) at the left costophrenic recess and lower-intensity "cooler" colors (yellow/green/blue) at the right base, supporting bilateral pleural effusion. Additional activation tracks the cardiac silhouette (cardiomegaly) and basilar opacities labeled as atelectasis and/or consolidation, likely reflecting dependent or compressive change in the setting of pleural fluid.

A posteroanterior chest radiograph was requested by the attending physician, obtained, and automatically subjected to AI-assisted interpretation. In the present case, the software flagged patterns compatible with atelectasis, cardiomegaly, consolidation, fibrosis, and pleural effusion, the latter being suggested as bilateral as described in Figure 1 panels A-C.

The posteroanterior chest radiograph above demonstrates preserved pulmonary transparency without focal alveolar consolidation. The mediastinum and hila show no significant abnormalities. The cardiac silhouette appears enlarged with regular contours. A pleural effusion is evident on the left, with a classic meniscus sign as follows: a curved upper margin concave upward on the upright chest X-ray, reflecting gravity-dependent layering with fluid adhesion to pleural surfaces and surface tension causing a greater lateral extent along the chest wall.

On the right side, the hemidiaphragm appears elevated, with a less defined and flattened contour, associated with an ill-defined costophrenic angle without a typical meniscus, findings consistent with a possible subpulmonic effusion [19]. Although the right-sided pleural fluid was ultimately characterized as possible predominantly subpulmonic, its frontal radiographic appearance deviated from the classic "textbook" pattern. In routine teaching examples, a subpulmonic effusion typically produces a smooth, apparently elevated hemidiaphragm with a lateral peak and blunting of the costophrenic angle. In this case, those signs were comparatively subtle, likely because the right pleural fluid was distributed mainly in the posterior/subpulmonic recess rather than forming a prominent lateral meniscus, and because the more conspicuous contralateral effusion drew attention away from the right base. Overall, the findings are most consistent with bilateral pleural effusions, with the left greater than the right.

In Figure 1 panel B, the AI ellipse overlay highlighted pleural effusion as the most relevant abnormality, with higher confidence on the left than on the right, supporting bilateral involvement and possible right subpulmonic underestimation. In Figure 1 panel C, the AI heatmap overlay (saliency map) highlights regions that contribute to the model output. Warmer colors (red/orange/yellow) indicate higher model attention, while cooler colors (green/blue) indicate lower attention. Predominant basal activation, stronger on the left than on the right, is consistent with bilateral pleural effusion, with comparatively lower right basal activation in keeping with a possible subpulmonic distribution. Accordingly, the AI-assisted analysis flags findings compatible with bilateral pleural effusion, atelectasis, basilar consolidation, cardiomegaly, and a right upper lung field fibrosis pattern.

In both Figure 1 panels B and C, the system does not highlight calcification, mediastinal widening, pulmonary nodules, pneumothorax, or pneumoperitoneum, which are shown as low-likelihood outputs in this case. The tuberculosis (TB) screening output is provided as an additional pattern-based assessment and does not establish a diagnosis.

Given the suspicion of additional right-sided subpulmonic fluid, the radiographer trained in screening ultrasound complemented the study with a focused assessment of the pleuro-diaphragmatic transitions, confirming an anechoic right subpulmonic pleural effusion (Video 1 panels A-C).

**Video 1:** Right pleuro-diaphragmatic transition ultrasound (panels A-C). (A) Longitudinal intercostal view using the liver (LI) and right kidney (RK) as landmarks, showing an anechoic pleural effusion (PE) above the diaphragm. A positive spine sign is present, with vertebral bodies visualized cranially beyond the diaphragm, consistent with enhanced ultrasound transmission through pleural fluid. (B and C) Sequential right upper quadrant sweeps (including a more cranial plane) demonstrate a clearly visualized suprahepatic vein within the liver and a sharply delineated, bright (hyperechoic) diaphragmatic contour, with persistent anechoic fluid above the diaphragm, confirming pleural effusion.

In the right upper quadrant views, the liver and right kidney served as anatomical landmarks. Pleural fluid was identified above the diaphragm, with a positive spine sign (Video 1 panel A) [20]. In a second plane, a hepatic (suprahepatic) vein was clearly visualized within the liver; the diaphragm was sharply delineated as a hyperechoic curvilinear interface, and an anechoic fluid layer was evident above the diaphragm, compatible with pleural effusion (Video 1 panel B). In an alternative, more cranial plane, the hepatic vein remained identifiable, the diaphragm was well defined, and a distinct anechoic collection was again seen above it, confirming pleural effusion (Video 1 panel C). Blunting of the left recess study through sonography is depicted in Video 2 panels A-C.

**Video 2:** Left pleuro-diaphragmatic transition ultrasound demonstrating pleural effusion (panels A-C). (A) Longitudinal left intercostal view obtained posterior to the mid-axillary line, using the spleen (SP) as an abdominal landmark; an anechoic pleural effusion (PE) is seen above the diaphragm with adjacent compressed/atelectatic lung (LU) with static air bronchograms. (B and C) Progressively more anterior intercostal sweeps in a near-axial (transverse) orientation, better depicting the anterior component of the pleural effusion and its cranioventral extension along the pleural space.

Here, the pleural effusion is seen in a longitudinal intercostal view acquired posterior to the mid-axillary line (Video 2 panel A), using the spleen as an abdominal landmark, showing an anechoic pleural effusion above the diaphragm with adjacent compressed/atelectatic floating lung. Subsequently, progressively more anterior intercostal sweeps in a near-axial (transverse) orientation (Video 2 panels B and C) better depicted the anterior component of the effusion. The patient was subsequently treated with inhaled bronchodilators, specifically ipratropium bromide (0.25 mg/mL) and salbutamol (5 mg/mL), both administered via nebulization.

**Figure 2:**
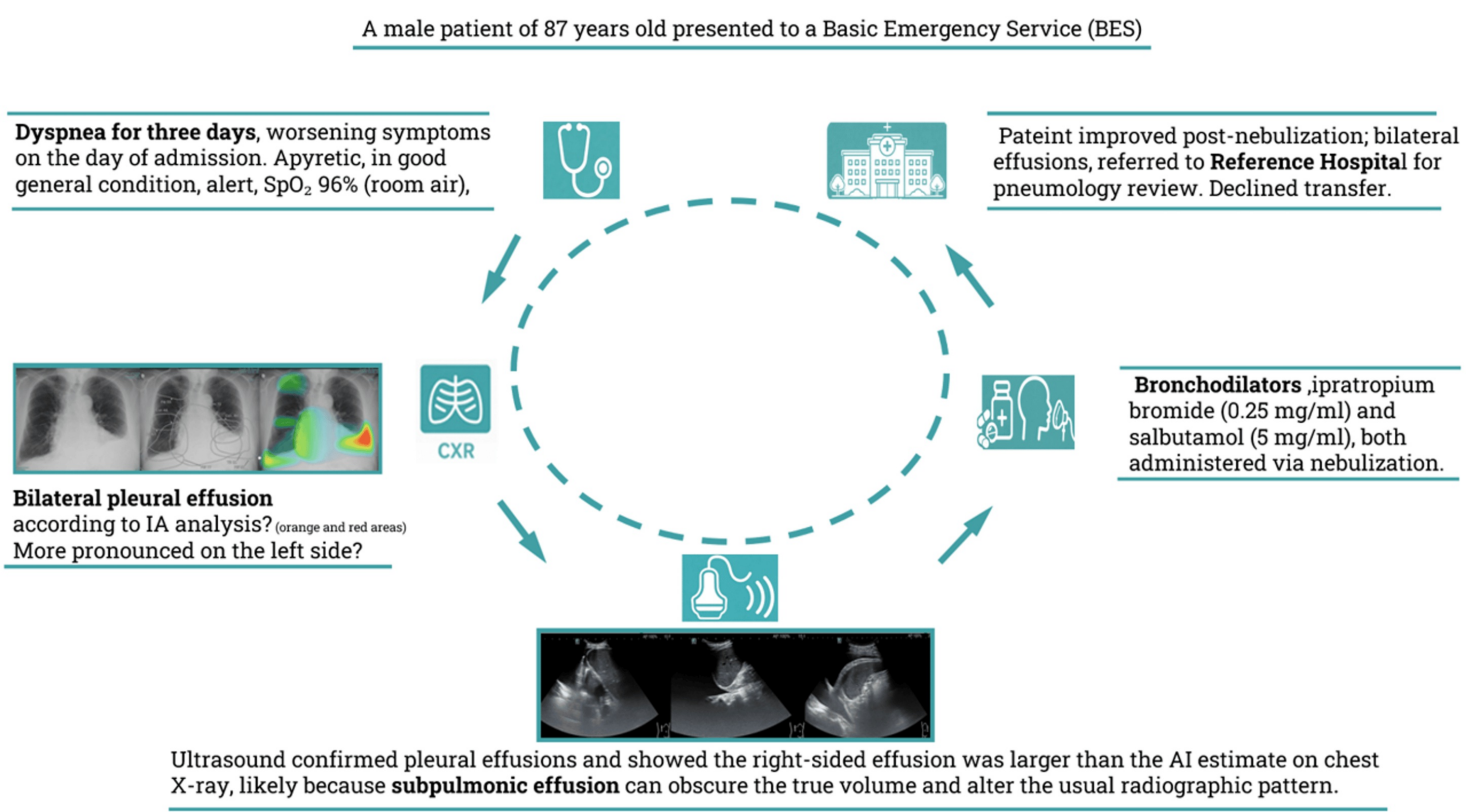
A summary of diagnostic timeline, key imaging-based decisions, and final referral. An overview of the diagnostic sequence and resulting management steps is presented here, integrating chest radiography, screening sonography, and subsequent referral.

Despite medical advice and the indication for transfer to a referral hospital for further investigation and potential admission, the patient refused. He signed a discharge form against medical advice and left the facility. Figure 2 provides an iconographic summary of the diagnostic timeline, highlighting the sequence of imaging examinations, key conclusions from chest radiography and screening sonography, and subsequent management and referral.

## Discussion

In fact, the AI X-ray thoracic analysis system performed remarkably well by rapidly detecting bilateral pleural effusion, which was indeed the most significant pathological finding. We acknowledge that in this subtle presentation, the abnormality may have been overlooked by many human readers. Small or atypically distributed collections, particularly subpulmonic fluid, can appear deceptively minor on chest radiography, so we used screening ultrasound as the confirmatory step. Ultrasound verified pleural fluid and demonstrated a predominantly right-sided subpulmonic effusion that appeared greater than suggested by the radiograph, although it remained below a threshold that would prompt thoracentesis. Importantly, this discrepancy is consistent with known limitations of chest radiography for small or dependent effusions. Heffner et al. reported that supine chest radiography may miss up to 500 mL of pleural fluid, with sensitivities as low as 47-60% [21]. Even on upright posteroanterior radiographs, small to moderate effusions may remain occult. Both Ilsen et al. and Karkhanis and Joshi cite approximately 200 mL as the commonly quoted volume at which blunting of the lateral costophrenic angle typically becomes apparent on an upright posteroanterior film [22,23]. Ilsen et al. further emphasize that, because the posterior costophrenic sulcus is deeper, volumes up to approximately 500 mL, or even more, may occasionally be present without clear lateral costophrenic angle blunting, particularly when fluid layers posteriorly or assumes a subpulmonic distribution [22].

In contrast, thoracic ultrasound can detect very small pleural effusions and has consistently shown high diagnostic performance. Soni et al. note that effusions of approximately 20 mL are more reliably identified, with reported sensitivities of 92-100% and specificities of 93-100% across key studies. Likewise, Lichtenstein concluded that, based on pooled data, the sensitivity and specificity of thoracic ultrasound for pleural effusion are 92% and 93%, respectively [24,25].

It should be noted that detecting pleural effusions on ultrasound is inherently operator-dependent and relies on the examiner’s technical expertise, which represents an important limitation of the modality. These findings strongly support the role of ultrasound as an essential complement to chest radiography, particularly in cases of subpulmonic or atypical effusions, where plain films may underestimate the presence and true volume of the effusion.

AI results should be interpreted alongside expert human review and clinical correlation, given their potential added value in subtle cases, and confirmed when appropriate with complementary imaging, such as thoracic ultrasound to verify and characterize the pleural effusion, including a potential subpulmonic component.

## Conclusions

This case illustrates a practical, stepwise approach to an equivocal chest radiograph in a peripheral emergency setting. The AI system flagged pleural effusion as the most relevant abnormality, supporting suspicion and prioritization when the radiographic finding was subtle. However, atypical distributions, such as subpulmonic fluid, can limit pattern-based systems' ability to reliably estimate extent, and we acknowledge that size estimation from CXR alone is inherently challenging. For this reason, screening ultrasound was used as the confirmatory step to verify the presence of the effusion and better characterize it, providing the level of certainty needed to guide disposition decisions. The key message is therefore not that AI should be used to quantify effusion size, but that AI can support detection and triage when CXR is not clear, while bedside screening ultrasound remains the complementary tool to confirm and characterize pleural fluid in real time.
